# Large Scale Study of Ligand–Protein Relative Binding Free Energy Calculations: Actionable Predictions from Statistically Robust Protocols

**DOI:** 10.1021/acs.jctc.1c01288

**Published:** 2022-03-16

**Authors:** Agastya P. Bhati, Peter V. Coveney

**Affiliations:** † Centre for Computational Science, Department of Chemistry, University College London, London WC1H 0AJ, United Kingdom; ‡ Informatics Institute, University of Amsterdam, P.O. Box 94323, 1090 GH Amsterdam, Netherlands

## Abstract

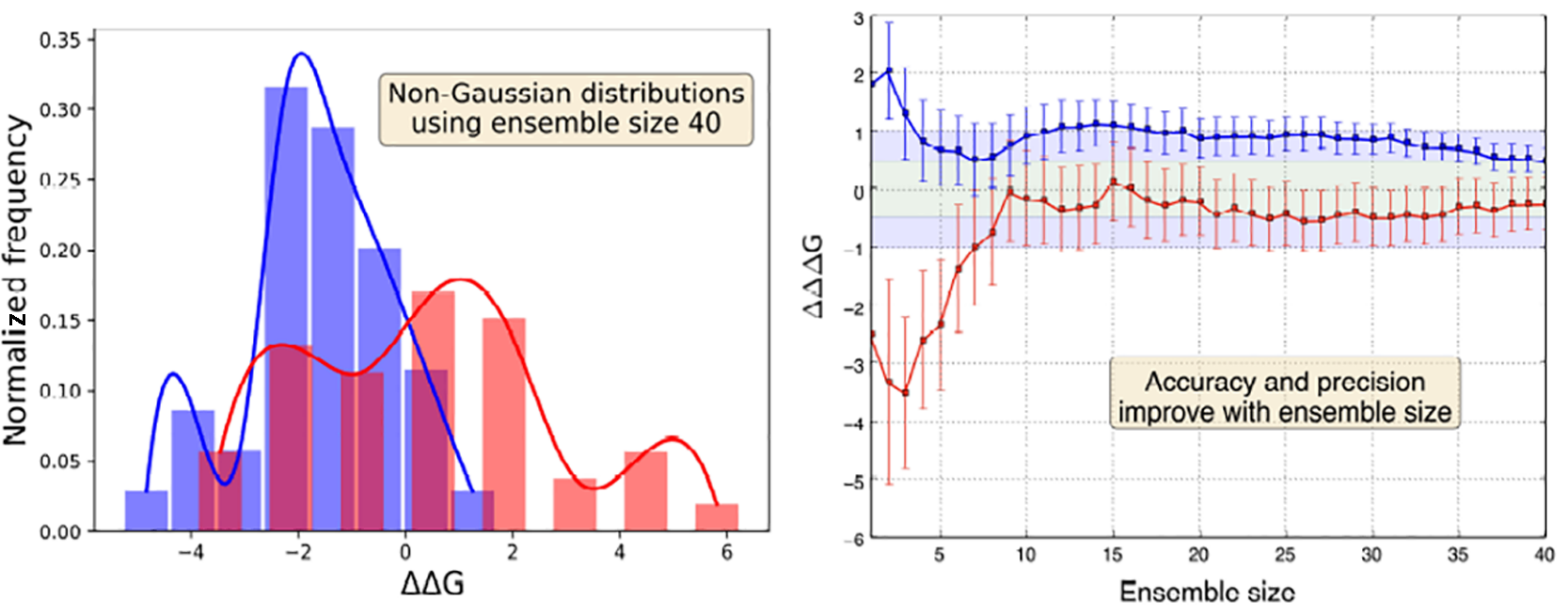

The accurate and reliable prediction of protein–ligand binding affinities can play a central role in the drug discovery process as well as in personalized medicine. Of considerable importance during lead optimization are the alchemical free energy methods that furnish an estimation of relative binding free energies (RBFE) of similar molecules. Recent advances in these methods have increased their speed, accuracy, and precision. This is evident from the increasing number of retrospective as well as prospective studies employing them. However, such methods still have limited applicability in real-world scenarios due to a number of important yet unresolved issues. Here, we report the findings from a large data set comprising over 500 ligand transformations spanning over 300 ligands binding to a diverse set of 14 different protein targets which furnish statistically robust results on the accuracy, precision, and reproducibility of RBFE calculations. We use ensemble-based methods which are the only way to provide reliable uncertainty quantification given that the underlying molecular dynamics is chaotic. These are implemented using TIES (Thermodynamic Integration with Enhanced Sampling). Results achieve chemical accuracy in all cases. Ensemble simulations also furnish information on the statistical distributions of the free energy calculations which exhibit non-normal behavior. We find that the “enhanced sampling” method known as replica exchange with solute tempering degrades RBFE predictions. We also report definitively on numerous associated alchemical factors including the choice of ligand charge method, flexibility in ligand structure, and the size of the alchemical region including the number of atoms involved in transforming one ligand into another. Our findings provide a key set of recommendations that should be adopted for the reliable application of RBFE methods.

## 1 Introduction

With the increasing power of supercomputers, the use of computational methods in the field of drug discovery has risen rapidly. *In silico* methods can support the identification of potential therapeutics by accelerating the process of screening vast real or virtual libraries of chemical compounds and/or *de novo* structures based on their binding strength to a given target protein.^1^ The binding affinity, also known as the binding free energy, is a quantitative measure of the strength of ligand–protein binding. Thus, computational methods for predicting binding free energies of ligand–protein complexes can play an important role in drug discovery. The average cost and time required to develop one drug stand at over $2 billion and 10 years, respectively.^2,3^ Improving the reliability of such methods should substantially reduce the time and cost associated with bringing novel drugs to market. The urgent need to dramatically accelerate the process of drug discovery has been made manifest during the COVID-19 pandemic.

Relative binding free energy (RBFE) methods based on classical molecular dynamics, that enable accurate prediction of protein–ligand binding affinities, offer an attractive route to optimize ligand–protein interactions on the drug discovery pathway.^4^ They can also be useful in personalized medicine.^5^ However, historical challenges such as high computational costs and consequent lack of sufficient sampling to obtain statistically robust results, force field accuracy, and time to solution as well as technical challenges in setting up and performing such calculations have limited the successful application of RBFE methods.^6^ Several recent advances in software as well as hardware along with methodological improvements have provided a boost to these methods in terms of their applicability, especially in drug discovery.^7^ Improvements in force field and ligand parameters, growing efficiency of high performance computing resources, the advent of GPU accelerators and codes compatible with them have all made valuable contributions. Automation tools are also now available for a quick and easy setup of RBFE calculations.^8−12^ For instance, the FEP+ package^8^ introduced a few years ago shrink-wraps the entire process of the RBFE setup, providing an impressive user-friendly interface for such calculations. Its expense and proprietary nature have restricted access to large pharmaceutical companies as well as its scientific evaluation.

Our group has publicly released the TIES toolkit^13^ to automate the process of setting up, running, and analyzing RBFE calculations using the ensemble simulation-based alchemical approach named Thermodynamic Integration with Enhanced Sampling (TIES).^14^ It consists of two components, TIES20 and TIES-MD. The former can be used to prepare TIES input by automatically identifying appropriate ligand mapping and building hybrid ligand molecules based on the TIES approach. The latter can be used to perform calculations and analyze results. The TIES toolkit thus provides a direct route for anyone interested in quickly setting up and executing RBFE calculations free of charge.

Another major issue is the lack of reproducibility and control of uncertainty in such methods due to the extreme sensitivity of classical molecular dynamics (MD) simulations to the initial conditions given their chaotic nature.^15^ This is manifested in the fact that two independent MD trajectories diverge exponentially with time and explore very different microstates. It is this behavior that confers the “mixing” ergodic property on MD simulation, a property stronger than ergodicity which is required to guarantee that the state of thermodynamic equilibrium may be reached.^15^ While one-off simulations, no matter how close their initial conditions in phase space, produce different results each time they are run, ensembles of such simulations produce a distribution of results whose properties (typically moments of the distribution including mean, variance, and so on) are statistically robust. Remarkably, the far reaching impact of chaos in MD has not been widely recognized. The book by Leimkuhler and Matthews^16^ is notable because it does pay attention to chaotic behavior, although it does not address the impact of dynamical chaos on uncertainty quantification. The only way to deal with this feature is to use ensemble-based approaches which ensure the statistical reproducibility of results.^14,15,17−24^ Although when we first advocated these methods we encountered resistance, it is noticeable that many practitioners now acknowledge that results based on one-off simulations may be grossly unreliable, and it is becoming more common to read of authors performing several “repeats” of calculations in order to estimate the uncertainty in their results (often without recognizing our previous work in this context).^6,26−79^ [In a private communication following the publication of Wan et al.,^22^ Schrödinger LLP agreed on the need for ensembles to produce statistically reliable results. More recently, they have acknowledged the need to properly handle uncertainties in their published article.^25^] For instance, Groot et al. perform 3 repeats in their recent free energy estimation studies.^26−28,79^ On the other hand, the same authors (the set of people common in all these studies) advocate 20 repeats when studying the effect of box sizes on thermodynamic properties^30^ which betrays a lack of consistency and systematic approach in addressing uncertainty estimation.

Indeed, for not unrelated reasons we are still not at a stage where RBFE methods can be routinely applied with confidence in the pharmaceutical industry let alone by clinicians to predict drug resistance in personalized medicine.^5^ There are many associated factors that need to be addressed in order to ensure that these methods can be employed in a routine fashion by end users. Quality of force field and ligand parameters, choice of alchemical region and the topology scheme (single, dual or mixed), handling of charge-changing transformations, protein starting structure, ligand pose placement, tautomerization, and ionization states are all issues that affect the quality of results.

There are a few recent publications that describe “best practices” for alchemical free energy methods and discuss some of the issues mentioned above.^6,7^ However, the major shortcoming of such articles is that their proposed guidelines are not based on statistically robust analysis of free energy predictions. For our part, we are interested in rendering such simulations actionable; this requires predictions to be accompanied by full uncertainty quantification.

However, there are three published works (including a very recent one) that include a few hundred ligand transformations studied using alchemical relative free energy methods. The purpose of these papers is to demonstrate the applicability of their RBFE methods.^8,26,79^ Neither the aforementioned best practice articles nor these large scale studies furnish a systematic analysis of the way in which the factors mentioned affect RBFE predictions.

Here, we address these lacunae by performing a statistically robust analysis of RBFE predictions for a large data set comprising 503 ligand transformations spanning 305 ligands and 14 target proteins covering a broad range of molecules and targets relevant for medicinal chemists and provide definitive recommendations concerning the protocols to use that can deliver actionable predictions.

We should emphasize here that the use of TIES does not restrict the validity of our findings. They are true for any RBFE method including, in particular, the so-called free energy perturbation (FEP) approach. Indeed, we have conducted a number of studies which confirm the general power of the approach and agreement with other methods within an ensemble-based approach.^17,22^ Moreover, in a recent study, we show that ensemble-based FEP and TIES can be performed concurrently at little extra cost, and their results will be statistically identical.^31^

The same general behavior is also exhibited by nonequilibrium MD methods. Indeed, this was reported by Potterton et al.^32^ where it was found that an ensemble size of 10 was necessary to reliably predict relative residence times for ligands. This is equally applicable to other nonequilibrium methods including free energy methods such as those based on Jarzynski’s inequality.^26,33,34^

Similarly, machine learning (ML) techniques are increasingly being employed in the field of free energy predictions.^35^ Of particular interest are studies combining ML and physics-based methods to accelerate free energy predictions for the selection of potential therapeutics.^36−39^ Such approaches have considerable potential and may be applicable to binding free energy predictions too, but they have many of their own limitations that need to be overcome. One major limitation is that ML methods are heavily data dependent, and hence, unless the data distributions of experimental data are understood and accounted for, such methods produce overconfident predictions.^40−42^ These methods are beyond the scope of the present paper and will not be discussed further here.

## 2 Scope of the Study

In this section, we briefly describe the various aspects of alchemical free energy methods that we have focused on in the present study. We summarize the prevailing view captured by best practice articles and comment on the open issues in this domain.

### 2.1 Ensembles and Distributions

It is an implicit assumption in almost all papers other than our own that the distribution of RBFEs obtained from different replicas in an ensemble is Gaussian. It is why, even if just 2 or 3 replica simulations are performed, those are deemed sufficient. Paliwal et al. claimed that hydration free energy distributions are Gaussian and deviate from this behavior at the 95% confidence level only when the ensemble sizes are increased above 110.^43^ However, in addition to the fact that their study is based on toy models with simple interactions, the dependence of normality on the ensemble size that they report raises questions as to the reliability of their interpretation of the general nature of free energy distributions. We have repeatedly shown that such distributions deviate from normality for complex protein–ligand systems.^19,23,44,45^ Moreover, this is not just confined to ligand–protein interactions but valid for many nonlinear systems with long-range interactions.^45,46^ Furthermore, a recent paper of ours demonstrates that the influence of the random number seed used in each MD simulation completely dominates the uncertainty accruing from the uncertainty in the parameters used to perform such simulations.^23^ The nature of free energy distributions associated with MD simulations and their important consequences are simply not touched upon by any of the publications discussed in the Introduction.

In the present study, we build upon our previous work on uncertainty quantification in free energy methods using ensembles to ensure robustness and reproducibility of RBFE predictions^14,17−19,22,23,31,44,45^ and apply our methods to fully quantify the nature of free energy distributions. We again report the occurrence of non-normal distributions of free energies obtained using large ensembles. We also discuss some important consequences of this observation.

### 2.2 “Enhanced Sampling” Degrades Performance

Replica exchange with solute tempering (REST2)^47,48^ is an enhanced sampling method that involves “heating” a highly localized part of the solute and exchanging information across concurrent simulations being performed at different intermediate points along the alchemical path and which have different “effective temperatures”. It has been claimed that REST2 accelerates local sampling around the alchemical region and should either improve the results or leave them unchanged.^8^ However, we have previously reported that the blind application of this method can degrade the quality of free energy predictions.^18,22^ In the present study, we report a systematic and statistically robust assessment of REST2 that establishes beyond doubt the general validity of our findings.

### 2.3 Electrostatic Charge Methods for Ligands

AMBER force fields^49^ embrace two popular methods for deriving partial electrostatic charges for ligands, namely RESP^50^ and AM1-BCC.^51^ The former involves a restrained fit to an electrostatic potential calculated with quantum mechanical (QM) methods, while the latter is a cheaper method based on semiempirical calculations and bond charge corrections. AM1-BCC charges are generally expected to provide values closely mimicking the RESP charges calculated at the Hartree–Fock/6-31G* level of theory but can be obtained much faster. However, differences have been reported between results emanating from the two charge systems albeit using only a small data set with no clear conclusion as to which should be preferred.^52^ Here, we perform a comparison on a statistically significant data set and report robust and conclusive findings.

### 2.4 Modified Dual-Topology Scheme

The majority of implementations of the alchemical free energy methods rely on either a single- or a dual-topology scheme for performing the alchemical transmutations. The strict dual topology scheme requires duplication of the system with atoms corresponding to the two end-states present at all stages, albeit not interacting with each other. This makes it difficult to obtain converged results and requires the application of spatial restraints. On the other hand, the single topology implementation requires introducing “dummy” atoms and becomes trickier as the two molecules become increasingly chemically dissimilar.^80^ Specially noteworthy are cases which involve changing the sizes of rings.^53^ To overcome these issues, a hybrid single-dual-topology approach has been introduced.^54^ However, it has a complicated implementation (currently only available with NAMD) and remains to be tested and validated using a large data set. The modified dual topology scheme employed in our TIES protocol^14,19^ overcomes the above issues in both the single and the dual topology schemes. It has been widely validated in our previous work, and we demonstrate its success in the present study too.

### 2.5 Size of Alchemical Region

When employing topology schemes such as the hybrid single-dual introduced by Jiang et al. or our TIES modified dual topology scheme, there is an additional variable involved: the number of atoms in the alchemical region. Hereafter, we will refer to it as the size of the alchemical region. We have shown previously that the precision of our TIES results is inversely proportional to this quantity due to slow convergence.^14,19^ In addition, if the alchemical region so defined includes charged groups, then the error bars become even larger. In the present work, we provide further related observations and demonstrate successful ways to deal with such situations.

## 3 Theory

Thermodynamic Integration (TI) is a common alchemical method used for calculating free energies.^55,56^ It uses a control variable λ to define interactions between the two end states such that its lower and upper limits, 0 and 1, correspond to the initial and final states of the alchemical transformation studied. The free energy change corresponding to the said transformation is calculated using the following equation:
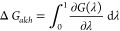
1It can be shown that
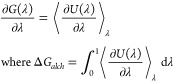
2where *U* is the potential energy of the system. It is worth mentioning that the above equation is only strictly valid in the thermodynamic limit, when both left and right sides of the equation are unique numbers. However, for finite systems with limited sampling of phase space, these quantities are stochastic variables with associated probability distributions.^14,23,44^ This is due to the extreme sensitivity of MD simulations to their initial conditions,^15^ which leads to differences in the configurations sampled for each repeat simulation and causes fluctuations in free energies obtained using eq 2.^14,17,18^ Therefore, when performing a single MD simulation at each intermediate λ state, every repeat calculation will yield a different result, which makes it unreliable. Thus, an ensemble simulation is necessary to bring such stochastic uncertainties under control. In this study, we employ an ensemble simulation-based method called “Thermodynamic Integration with Enhanced Sampling (TIES)” that involves performing an ensemble of MD simulations at each λ state and integrating the ensemble averaged energy derivative in eq 2 using stochastic calculus. The resultant free energies are reported along with proper estimates of associated aleatoric uncertainties. More details on TIES are available from prior publications.^14^ In a recent article, we show that, when employing ensemble simulations, the free energy predictions from TIES and FEP produce the same results within statistical error.^31^ Thus, ensemble simulations ensure reproducibility across free energy methods and extend the validity of our findings to other RBFE methods such as FEP and FEP+, and we expect the nonequilibrium TI approach.

The relative binding affinities for ligand–protein complexes can be calculated using a thermodynamic cycle with the following equations
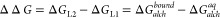
3where Δ*G*_L2(1)_ is the binding affinity for ligand L2(1), and  and  are free energy differences for alchemically transforming ligand L1 into L2 in protein and aqueous environments, respectively.

## 4 Methods

In this section, we describe the protein–ligand systems studied and provide details on the implementation of the various steps involved in the TIES protocol for calculation of free energies.

### 4.1 Data Set Studied

The ligand–protein systems used in this study are comprised of some selected benchmark sets that have already been studied with FEP+.^8,52,53,57−60^ This data set covers a wide range of ligands and target classes (305 ligands and 14 protein targets). We studied 503 ligand perturbations that include a wide range of chemical modifications typically seen in medicinal chemistry efforts. Out of the 14 protein systems studied here, 8 were part of the previous FEP+ study by Wang et al.:^8^ BACE, MCL1, TYK2, thrombin, CDK2, P38, PTP1B, and JNK1. The remaining 6 have appeared in subsequent FEP+ studies: PDE2,^57^ cMET,^58^ Galectin,^52^ and three additional BACE data sets.^53,59,60^ In addition, all of these systems except BACE (scaffold) were also studied using a nonequilibrium alchemical approach referred to as “PMX” hereafter in this article.^26^

### 4.2 TIES Approach

All free energy calculations in this study have been performed using the TIES protocol that has been described in detail in our prior publications.^14,19^ Briefly, it involves performing an ensemble simulation at each intermediate alchemical state followed by ensemble averaging of the bootstrapped potential energy derivatives so obtained. Such averaged energy derivatives are then integrated using the principles of stochastic calculus to get the final free energy difference along with associated uncertainties using eq 2. The standard protocol is to perform an ensemble of 5 MD simulations at each λ window of length 4 ns and uses 13 λ-windows as follows: 0, 0.05, 0.1, ..., 0.9, 0.95, 1. It should be noted that these standard settings for the TIES protocol were derived through a systematic study of the dependence of accuracy and precision of the TIES predictions on all these parameters.^14^ However, it should be noted that these standard values may need to be adjusted in some cases to control errors/uncertainties.

Our approach uses a modified dual topology scheme.^14,19^ This scheme involves selecting the maximal common substructure (MCS) for a given ligand pair which is structurally and chemically identical between the two ligands within the thresholds defined. The standard thresholds used are a 0.1e difference between the two ligands for atomic charges of individual atoms in MCS as well as their sums. First of all, a structurally identical MCS is identified. Thereafter, its chemical identicality is ensured by iteratively removing atoms from it until the charge tolerance criteria are met. The simplest approach to do so is to remove the atom with the highest charge difference in each iteration. Alternative approaches are to prioritize the removal of terminal atoms or atoms bordering the alchemical region or both. Our automated TIES topology builder tries all these approaches separately and chooses the one yielding the largest MCS while achieving the charge tolerance criteria.^13^

MCS is represented with a unique set of atoms in the simulation-ready system, whereas the remainder of the ligand constitutes the alchemical region. The alchemical regions for ligands corresponding to λ = 0 and 1 states are named “disappearing” and “appearing” regions, respectively. In the simulation-ready model, both disappearing and appearing regions are connected to the unique MCS through single bonds. Thus, there is no need to use any position restraints in our simulations unlike the case with the standard dual topology scheme where the entire ligand is coupled/decoupled with the environment, nor does it require introducing any dummy atoms (that is atoms introduced to account for the imbalance in the number of atoms between the two ligands and which remain fully noninteracting with their neighboring atoms when the smaller of the two ligands is fully coupled with the environment) unlike their occurrence within the single topology scheme. The modified dual topology scheme used in TIES thus overcomes the drawbacks of both single and dual topology schemes.

Initial structures for the 14 proteins were taken from downloaded PDB data sets and aligned to those from previous FEP+ studies. For ligands, they were derived from the supplementary data provided with prior studies. All crystal water molecules within 5 Å of the protein were included. Protonation states and tautomeric forms for histidine residues were kept consistent with previous FEP+ studies. GAFF (v2)^61^ parameters were used to prepare ligand molecules with charges calculated using AM1-BCC model.^51^ The AMBER ff14SBonlysc^49^ force field was used to parametrize proteins. Our systems were solvated in an orthorhombic TIP3P^62^ water box with at least a 14 Å solvent in all directions. Sodium and chloride ions were used to neutralize the system electrostatically employing Joung-Cheatham ion parameters.^63^ AmberTools20^64^ was used to perform parametrizations and prepare all models.

For all transformations, the hybrid ligand in protein environment (referred to as “complex”) was first energy minimized, followed by a 20 ps NVT equilibration and a 2 ns NPT equilibration. Pressure and temperature were maintained at 1 atm and 300 K using a Berendsen barostat (compressibility of 4.57 × 10^–5^ bar^–1^ and relaxation time of 100 fs) and a Langevin thermostat (damping coefficient of 5 ps^–1^), respectively. The production run time was 4 ns. A time step of 2 fs was used. When a hybrid ligand in the water environment (referred to as “ligand”) was simulated, the entire protocol remained the same except that the NPT equilibration step was only 1 ns long. During both minimization and equilibration steps, protein backbone atoms were initially constrained to their initial positions and were slowly allowed to relax. Periodic boundary conditions were employed with long-range electrostatics handled by the Particle Mesh Ewald (PME) method.^65,66^ A nonbonded cutoff of 12 Å was used. The van der Waals interactions were smoothly switched off between 10 and 12 Å, being linearly decoupled/coupled between λ value 0 and 1 for disappearing and appearing atoms, respectively. The standard NAMD soft-core potential^67,68^ was used for the van der Waals terms with the radius-shifting coefficient of 5 to avoid singularities. Electrostatic interactions of the disappearing atoms were linearly decoupled from the simulations between λ values of 0 and 0.55 and completely turned off beyond that, while those of the appearing atoms were linearly coupled to the simulations from λ value 0.45 to 1 and completely extinguished otherwise. While the coordinates were recorded every 10 ps, energy derivatives with respect to λ were recorded every 2 ps. When the enhanced sampling REST2 protocol^47,48^ was employed, all alchemical atoms constituted the “hot” region in the ligand simulations, whereas all alchemical atoms along with all protein residues falling within 3 Å of the alchemical atoms constituted the “hot” region in ligand–protein simulations. The maximum “effective” temperature used was 600 K.

All simulations were performed with NAMD 2.14^69^ using up to 96 CPUs per MD simulation on SuperMUC-NG^70^ and up to 128 CPUs on ARCHER2^71^ as well as Theta.^72^ Some simulations were also performed on Summit using RADICAL CyberTools,^73^ such that CPUs were occupied for TIES simulations concurrently with GPUs being utilized for other calculations. A typical TIES calculation (5 MD simulations each at all 13 λ states) for complex and ligand systems required around 50k and 5k core hours on SuperMUC-NG, respectively. The wall clock time required to produce a ΔΔ*G* value for one transformation involving proteins of typical size (250–350 residues) is about 6–8 h using CPUs and  h using a single GPU. We note that ensemble-based workflows allow scale out on emerging exascale architectures so as to perform hundreds of ΔΔ*G* calculations within the same wall clock time as required for a single calculation. We have been able to exploit the entirety of SuperMUC-NG (ca. 310k cores) for such purposes several times in the past under so-called “block operations”.^74^

All hybrid ligands were built automatically using TIES20.^19,75^ The TIES toolkit has already been released for open use and can be accessed at https://www.ties-service.org.

## 5 Results and Discussion

**Figure 1:**
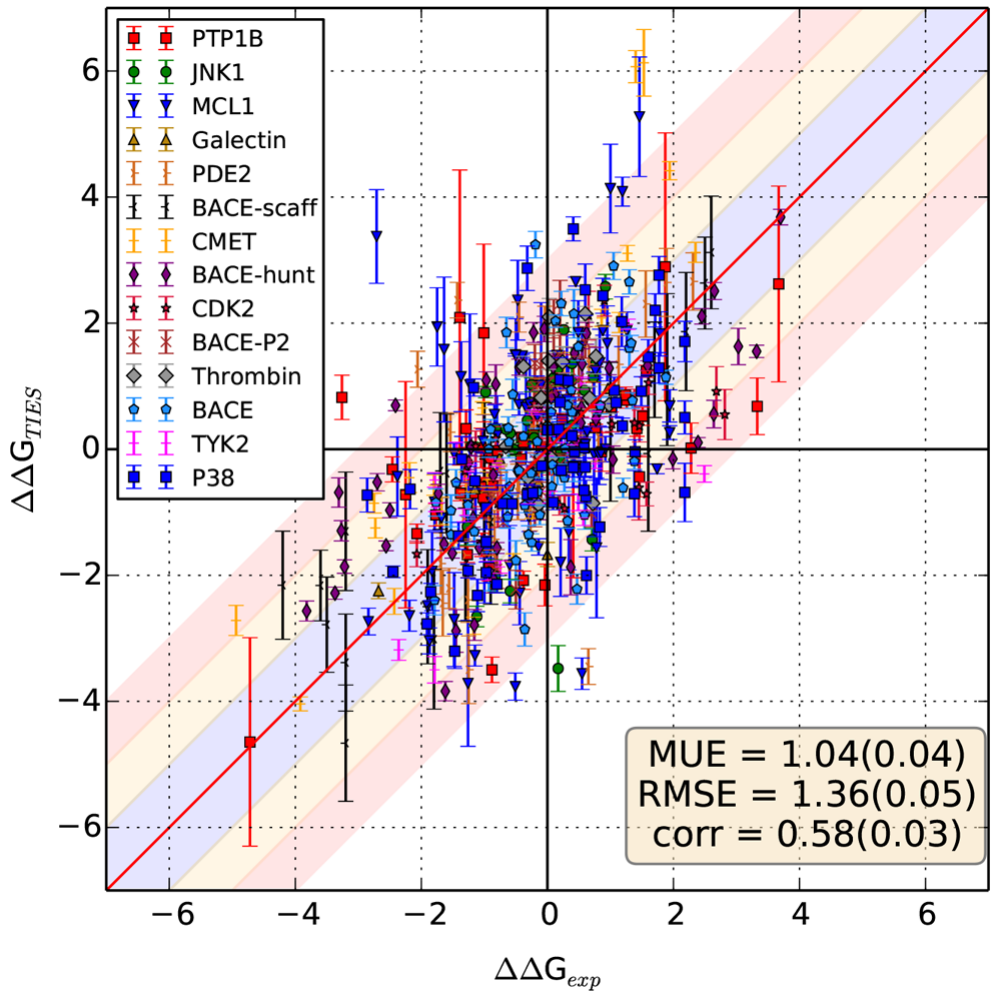
Correlation between experimental and calculated ΔΔ*G* values for all systems studied. The text box includes statistical measures of agreement–mean unsigned error (MUE), root mean squared error (RMSE), and Pearson correlation coefficient (corr) for the entire data set along with their standard errors in parentheses. The red line denotes the perfect correlation line (*y* = *x*), and the ±1, ±2, and ±3 regions have been shaded with different colors. All values are in kcal/mol. Error bars are not available for experimental data.

As noted previously, we have performed TIES calculations to estimate ΔΔ*G* values for 503 ligand pairs spanning 305 ligands bound to 14 different target proteins. The performance of systematic and extensive analysis using such a large data set makes our study unprecedented. A concise summary of the results obtained for the entire data set as well as for each protein system individually is shown in Table 1 and Figure 1. The mean unsigned error (MUE) and Pearson’s correlation coefficient (*r*_p_) for all 503 predictions when compared with corresponding experimental values are 1.04 ± 0.04 and 0.58 ± 0.03 kcal/mol, respectively. For individual protein systems, these values vary from 0.58 ± 0.18 to 1.41 ± 0.13 kcal/mol and from 0.24 ± 0.23 to 0.88 ± 0.04 kcal/mol for MUE and *r*_p_, respectively. Around 32%, 59%, 77%, and 87% of our predictions differ from the corresponding experimental ΔΔ*G*s by less than 0.5, 1, 1.5, and 2 kcal/mol, respectively. On the other hand, only 19 (∼4%) have experimental values differing by more than 3 kcal/mol, that is which fall outside the shaded regions in Figure 1. It is worth mentioning here that all these results have been obtained by a simple application of the standard TIES protocol. As we have mentioned, the TIES protocol is flexible, and hence the accuracy and/or precision of results can be improved by adjusting it in specific cases.^14^ We will show in the following sections that, on adapting the standard TIES protocol in various ways for different systems, one may further improve the reliability of the results yielding more robust ΔΔ*G* predictions.

**Table 1:** Summary of TIES Results^a^

system	ligand pairs	exp. range	RMSE	MUE	*r*_p_
BACE	58	–1.79 to 1.88	1.17(0.12)	0.91(0.10)	0.49(0.09)
BACE (Hunt)	60	–3.82 to 3.70	1.33(0.10)	1.10(0.10)	0.68(0.06)
BACE (P2)	26	–0.90 to 0.70	1.09(0.11)	0.92(0.11)	0.51(0.18)
BACE (scaffold)	21	–4.20 to 2.60	1.05(0.13)	0.87(0.13)	0.88(0.04)
CDK2	25	–2.07 to 2.81	1.15(0.13)	0.96(0.13)	0.43(0.15)
CMET	25	–4.94 to 2.35	1.80(0.30)	1.38(0.23)	0.84(0.04)
Galectine-3	7	–2.68 to 0.96	0.75(0.23)	0.58(0.18)	0.76(0.31)
JNK1	31	–1.27 to 0.92	1.23(0.17)	0.98(0.13)	0.45(0.17)
MCL1	71	–2.84 to 1.94	1.80(0.18)	1.41(0.13)	0.40(0.11)
P38	56	–2.86 to 2.18	1.26(0.12)	0.98(0.11)	0.59(0.08)
PDE2	34	–2.06 to 2.31	1.43(0.25)	0.95(0.18)	0.54(0.17)
PTP1B	49	–4.72 to 3.67	1.35(0.17)	0.99(0.13)	0.56(0.14)
thrombin	16	–0.66 to 0.98	1.07(0.15)	0.88(0.15)	0.24(0.23)
TYK2	24	–2.36 to 2.49	1.08(0.16)	0.88(0.13)	0.60(0.13)
					
All	503	–4.94 to 3.70	1.36(0.05)	1.04(0.04)	0.58(0.03)

a The number of alchemical transformations studied and corresponding values of several statistical parameters–root mean square error (RMSE) and mean unsigned error (MUE) for all TIES predictions as well as Pearson’s *r* (*r*_*p*_) between ΔΔ*G*_TIES_ and experimental results–are reported. Standard errors are included in parentheses. Exp. range denotes the range of experimental ΔΔ*G*s. The unit is in kcal/mol.

We compared RBFEs from FEP and TIES obtained using three different MD engines and found that our results were in agreement only when using ensemble simulations. This is not the case for results based on one-off simulations.^31^ Therefore, we expect to get the same results from ensemble-based FEP for this data set. Gapsys et al.^26^ recently reported the performance of a couple of other alchemical free energy methods (the nonequilibrium PMX and the proprietary shrink-wrapped FEP+) using most of this data set. A meaningful comparison of results from different methods and/or force fields is only possible when uncertainties associated with such calculations are under control.^31,44^ Gapsys et al. performed only 3 repeat calculations as opposed to using an ensemble approach while FEP+ manifests errors arising *inter alia* from the REST2 protocol (see section 5.6 and Wan et al.^22^)

### 5.1 Statistical Metrics

Utilizing the wealth of our large data set, we would like to emphasize an important point here which is often ignored in the literature. First of all, the range of ΔΔ*G*_exp_ values affects the prediction accuracy. The Pearson’s correlation coefficient is bound to be low for ligand pairs with experimental relative free energy differences less than 1 kcal/mol as evident from the data in Table 2 for both the entire data set as well as the subset with |ΔΔ*G*_TIES_ – ΔΔ*G*_exp_| < 1 kcal/mol. It can be clearly seen that *r*_p_ for the subset of ligand pairs with experimental relative free energy differences below 1 kcal/mol is always lower than the corresponding full data set as well as subsets with experimental values falling between 1 and 2, 2–3, and over 3 kcal/mol. This is true irrespective of the trend of their corresponding MUEs. This means that, for a narrow range of ΔΔ*G* values, smaller MUEs do not always translate into higher correlation and *vice versa*. From Table 1, we can see that thrombin, BACE (P2), and JNK1 have a narrow range of experimental ΔΔ*G*s, and all of them have low *r*_p_ values despite relatively small MUEs. Gapsys et al.^26^ also pointed out such inconsistencies between MUE and *r*_p_ for the thrombin data set using PMX and FEP+. Such trends in *r*_p_ make it a less reliable tool for quantifying results for a data set which comprises close to 60% of ligand pairs with experimental |ΔΔ*G*| values below 1 kcal/mol, such as the one used in this study and as is the case in the majority of studies. Furthermore, it should be noted that MUE values are bound to have associated statistical uncertainty of similar magnitude as those associated with individual ΔΔ*G* values ( kcal/mol). This means that, for ligand pairs with |ΔΔ*G*| < 1 kcal/mol, the percentage fluctuation in MUE values will be large, rendering them a less robust metric. In other words, since ΔΔ*G* predictions have associated uncertainties, MUEs will inevitably fluctuate by a similar amount as the predictions themselves. The key point is that performing one-off simulations or too few replicas is not robust, since there is always a substantial probability of making unreliable predictions.

**Table 2:** Number of Pairs of Transformations, MUE, and *r*_p_ for TIES Predictions with Ligands Pairs Categorized Based on the Range of Experimental ΔΔ*G* Values^a^

	all			|ΔΔΔ*G*| < 1		
|ΔΔ*G*_exp_|	no. of pairs	MUE	*r*_p_	no. of pairs	MUE	*r*_p_
< 1	300	0.92(0.04)	0.35(0.05)	195	0.49(0.02)	0.64(0.04)
≥ 1 and < 2	145	1.13(0.08)	0.65(0.04)	80	0.51(0.03)	0.92(0.01)
≥ 2 and < 3	38	1.48(0.20)	0.64(0.11)	16	0.41(0.06)	0.98(0.01)
≥ 3	20	1.47(0.22)	0.85(0.08)	5	0.22(0.11)	0.99(0.01)
						
total	503	1.04(0.04)	0.58(0.03)	296	0.48(0.02)	0.89(0.01)

a |ΔΔΔ*G*| denotes the absolute difference between experimental and calculated ΔΔ*G* values. Standard errors are included in parentheses. All values are in kcal/mol.

### 5.2 Uncertainties in Experimental Data

An important issue that is rarely taken into consideration is the uncertainty associated with experimental ΔΔ*G* predictions and its consequences. The experimental predictions of relative free energy differences have statistical uncertainties that often go unreported as is the case here and the vast majority of such publications.^25^ A direct consequence of this is a phenomenon called regression dilution that is well-known in statistics.^76^ It means that uncertainty in the known variable (ΔΔ*G*_exp_ in this case) causes biasing of the linear regression slope toward zero. This phenomenon has been reported in more detail in a recent publication where different statistical methods are used to demonstrate the biased least-squares regression slope and the extent of underestimation in predicted values has been quantified.^25^ It is possible to correct for such deviations when both the known and unknown variables are normally distributed, but in the case of free energy predictions (whether calculated or experimental), the distributions are not guaranteed to be normal (see section 5.3). This in turn means that linear regression models (*r*_p_) are not the best metric for quantifying the accuracy of free energy predictions, especially when the experimental results have large variances.^25^ Moreover, uncertainties in experimental predictions also undermine the reliability of MUEs when these values are small in magnitude (less than 1 kcal/mol).

### 5.3 Free Energy Distributions

**Figure 2:**
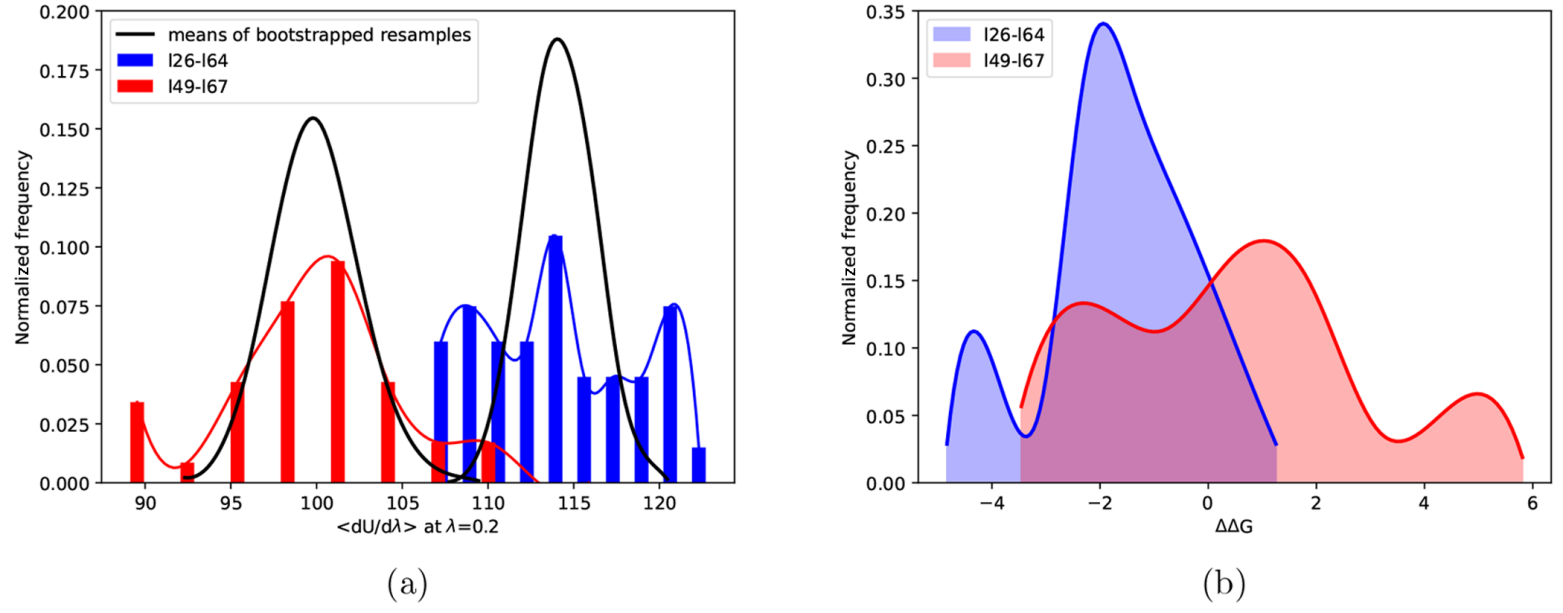
(a) Distributions of potential energy derivatives and their corresponding means of bootstrapped resamples for two MCL1 ligand pairs using 40 replicas per λ window; the distributions of energy derivatives (shown in blue and red as bar and solid line plots) exhibit non-normality, whereas the distributions of means of resamples of size 5 obtained using bootstrapping (shown in black as solid line plots) are normal owing to the central limit theorem. (b) Distributions of ΔΔ*G* values for the same two ligand pairs (shown in blue and red as solid line plots with the underlying area shaded).

It has been reported in numerous published studies^14,15,17−19,23,31,44,45,77^ that MD-based free energies are sensitive to their initial conditions. Thus, we get a distribution of free energies on performing ensembles of MD simulations with identical input except their starting velocities due to the aleatoric uncertainty in MD. We have shown that aleatoric uncertainty dominates all other forms of uncertainty and that ensemble simulations are essential.^23^ Parametric uncertainty is dampened between input and output, further exacerbating the role of chaos.^23^ An additional source of systematic error for chaotic systems originates from the use of floating point numbers.^78^ This is true irrespective of the free energy method used including the alchemical ones. Figure 2 displays the distributions of both potential energy derivatives at an arbitrary λ window as well as ΔΔ*G* values for a couple of ligand pairs bound to MCL1 using 40 replicas per λ window. It can be seen that  varies by up to 20 kcal/mol and ΔΔ*G* values by up to 10 kcal/mol across replicas for the chosen cases. Given the wide spread of these distributions, it is essential to perform ensemble simulations (often  replicas) in order to control the aleatoric uncertainty associated with the ΔΔ*G* estimations making the results reproducible. This can be demonstrated more clearly with the data in Table 3 where results using a single replica have been compared with those using ensemble simulations for the entire data set as well as various subsets of it. The accuracy of ensemble simulations is substantially better than any single replica, irrespective of the data set taken. For instance, the MUE for the entire data set using a single replica ranges from 1.19 ± 0.05 to 1.24 ± 0.05 kcal/mol, whereas it is 1.04 ± 0.04 kcal/mol for TIES. The corresponding numbers for the subset |ΔΔ*G*_exp_| < 1 kcal/mol are 1.00 ± 0.05 to 1.06 ± 0.05 kcal/mol versus 0.92 ± 0.04 kcal/mol. Similar trends are observed for *r*_p_ values. It is worth highlighting that the number of ligand pairs with |ΔΔΔ*G*| < 1 kcal/mol (that is, an absolute difference between experimental and predicted relative free energies of less than 1 kcal/mol) using ensemble simulations is higher than those using a single replica (296 versus 262–276). Thus, ensemble simulations yield binding free energy predictions for 20 to 34 more ligands with accuracy better than the 1 kcal/mol mark compared to one-off simulations.

**Table 3:** Replicawise Variation in Results^a^

	all		|ΔΔ*G*_exp_| < 1		|ΔΔΔ*G*| < 1		
replica	MUE	*r*_p_	MUE	*r*_p_	no.	MUE	*r*_p_
1	1.24(0.05)	0.48(0.04)	1.06(0.05)	0.29(0.06)	262	0.50(0.02)	0.88(0.01)
2	1.21(0.05)	0.47(0.04)	1.04(0.05)	0.29(0.06)	272	0.47(0.02)	0.89(0.01)
3	1.20(0.05)	0.50(0.04)	1.00(0.05)	0.34(0.06)	272	0.48(0.02)	0.88(0.01)
4	1.20(0.05)	0.53(0.04)	1.01(0.05)	0.32(0.05)	269	0.47(0.02)	0.89(0.01)
5	1.19(0.05)	0.52(0.04)	1.01(0.05)	0.29(0.05)	276	0.47(0.02)	0.89(0.01)
							
TIES	1.04(0.04)	0.58(0.03)	0.92(0.04)	0.35(0.05)	296	0.48(0.02)	0.89(0.01)

a Replica X denotes results obtained by taking values corresponding to the replica X of each λ window for all ligand pairs, whereas TIES means using the data from all 5 replicas to obtain ΔΔ*G*. “All” corresponds to the entire data set. Two subsets have also been included–one with experimental ΔΔ*G*s less than 1 kcal/mol and the other one with the difference between predicted and experimental values less than 1 kcal/mol. MUE is the mean unsigned error, and *r*_p_ is the Pearson’s correlation coefficient. Standard errors are included in parentheses. All values are in kcal/mol.

**Figure 3:**
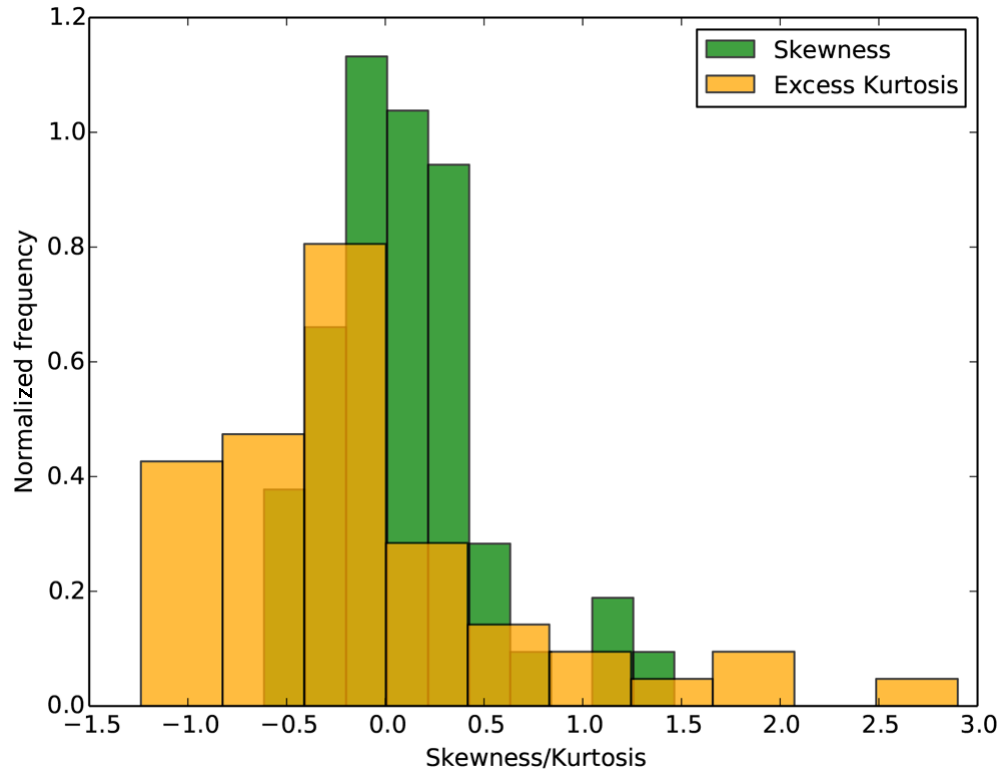
Distributions of skewness and excess kurtosis for ΔΔ*G* distributions of 51 different ligand pairs. Data for some ligand pairs have been taken from Bieniek et al.^19^

Another important observation from Figure 2 is the non-normality of the distributions of energy derivatives as well as the relative free energy differences. To further substantiate this point, we chose 51 different ligand pairs and performed ensemble simulations comprising 20 to 40 replicas at each λ window and obtained ΔΔ*G* distributions like the ones shown in the right panel of Figure 2. Skewness and excess kurtosis coefficients for each of these 51 ΔΔ*G* distributions were calculated. Figure 3 displays distributions of the skewness/excess kurtosis coefficients so obtained. Nonzero skewness coefficients indicate asymmetry that favors higher frequency measurements away from the mean. On the other hand, many of the ligand pairs have positive excess kurtosis indicating the abundance of outliers. We obtain non-normal distributions of predicted binding affinities similar to those displayed in Figures 2 and 3 even on extending the ensemble size up to 135 replicas for a few of these 51 ligand pairs (data yet to be published). It is evident that one could never understand the behavior of these ensembles by only running 1−3 replicas. We found that the distribution of experimental binding free energies obtained from a limited number of selected ligands which have been subjected to many repeated measurements over a lengthy period are also non-Gaussian.^81^ The underlying implication of non-normal statistics is that more frequent occurrence of outliers means larger error bars and that graphs comparing predicted and experimental predictions will deviate from ideal linear plots with all points lying close to a straight line with slope 1. This needs to be borne in mind when interpreting the resultant correlation plots. Statistical tools like bootstrapping and linear regression should still, in principle, be applicable. However, their quantitative reliability for non-normal distributions is questionable for small sample size as the law of large numbers is not then applicable. It should also be noted that for a Gaussian distribution, more points should not change the expectation value, and the variance would reduce with the inverse square root of the number of points included. On the other hand, for an asymmetric distribution, the more points one includes the more reliable are both the mean and the variance. The variance for non-normal distributions usually converges far more slowly, so the fluctuations persist for much longer.^42^ This further supports the importance of performing ensemble simulations for MD-based methods and highlights that a small number of repeats is not sufficient.^42^

### 5.4 Precision and Accuracy Are Related

A unique feature of the ensemble methodology is that it requires adapting the ensemble size to improve the precision of results, which in turn leads to better accuracy in several cases. To further clarify this point, we picked out the worst performing MCL1 system from our data set. This protein system has something unique about it that makes it the worst performing not only with TIES but also with PMX while being the second worst with FEP+.^8,26^ This behavior is likely to be related to the flexible structure of a generic MCL1 ligand with its two ends (one hydrophobic, the other charged) connected with a 4-membered linker such that the hydrophobic end is buried deep into the lower pocket, while the charged end interacts with the positively charged arginine residue (R263). The phenomenon has been described in more detail in the Supporting Information of the study by Bhati et al.^14^ The intrinsic flexibility of this ligand leads to larger uncertainties in the predicted ΔΔ*G* values for many of the MCL1 ligand pairs.

**Figure 4:**
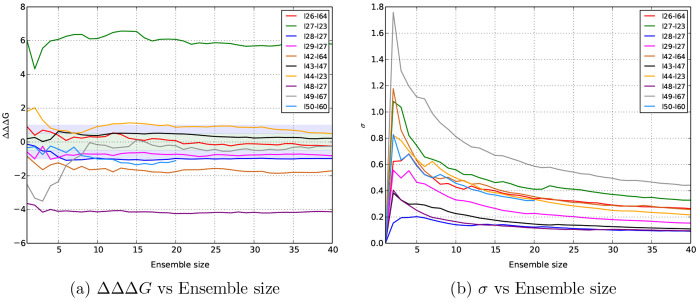
Variation of TIES results with the ensemble size for a selection of MCL1 ligand pairs. In (a), ΔΔΔ*G* refers to the difference between the predicted and experimental relative binding affinities, while in (b), σ denotes the standard error of a TIES prediction.

**Figure 5:**
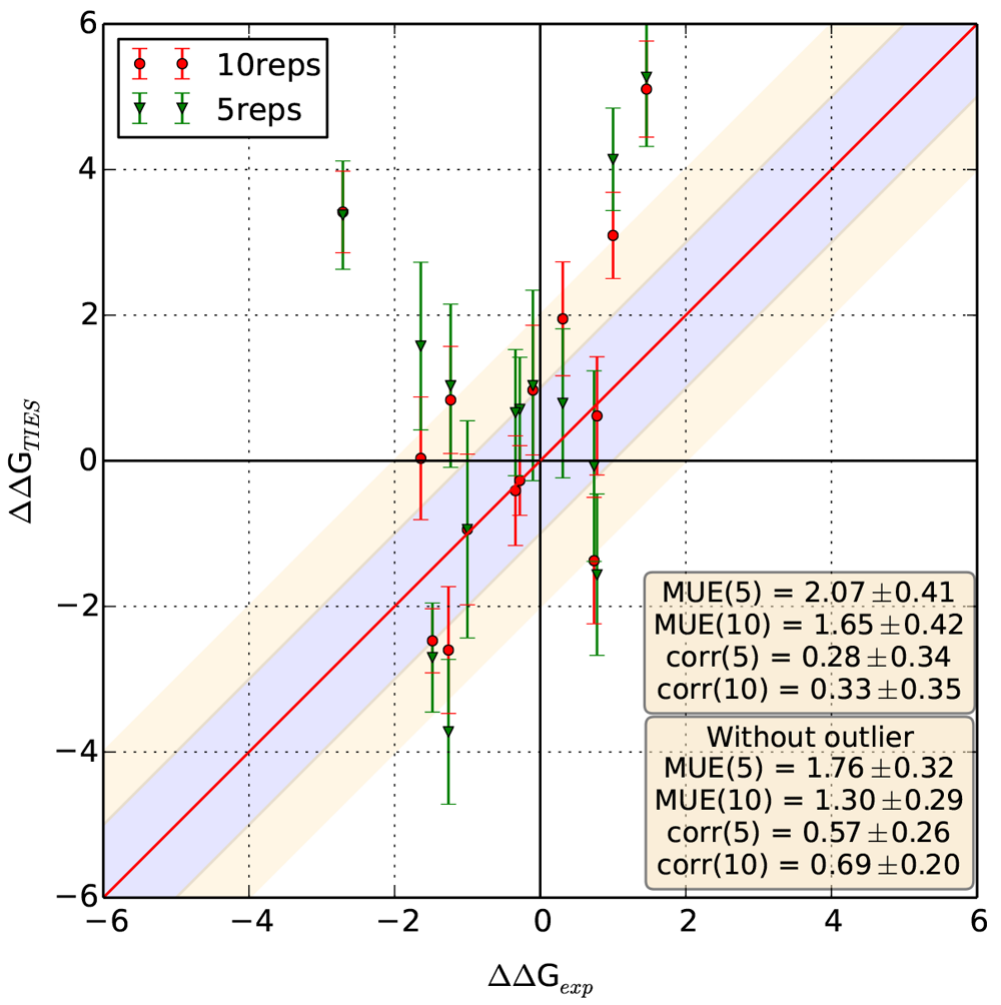
Effect of increasing the ensemble size on the accuracy of predictions for flexible ligand structures. We display a comparison between results using ensemble sizes of 5 and 10 for 14 MCL1 ligands pairs with the largest uncertainties. Statistical metrics along with associated standard errors are reported in text boxes in the bottom right corner.

Figure 4(b) shows the variation of TIES uncertainty with the ensemble size increased to 40 replicas for 10 MCL1 ligand pairs. It is clear that the uncertainty consistently drops as the ensemble size is increased for all these cases. A similar behavior has been reported in several studies.^19,31,44,45^Figure 4(a) exhibits the variation of ΔΔΔ*G* (that is ΔΔ*G*_TIES_ – ΔΔ*G*_exp_) for the same set of MCL1 ligand pairs. It is interesting to note that the two cases with the largest uncertainties (l49-l67 in gray and l44-l23 in orange) also have large ΔΔΔ*G* values. More interestingly, the accuracy improves for both these cases, as we increase the ensemble size. This suggests that better precision may lead to better accuracy in systems such as MCL1 that have flexible structures. To further investigate this, we selected all MCL1 ligand pairs with uncertainties  kcal/mol using the standard TIES protocol (ensemble size 5). There were 14 such ligand pairs for which we increased the ensemble size to 10. Figure 5 compares the results from ensemble sizes 5 and 10 for all these cases. We observe a substantial improvement in the overall accuracy for this set of 14 ligand pairs simply on increasing the ensemble size to 10 which can be attributed solely to better precision. MUE improves from 2.07 ± 0.41 to 1.65 ± 0.42 kcal/mol, and *r*_p_ increases from 0.28 ± 0.34 to 0.33 ± 0.35 kcal/mol. There is a clear outlier whose accuracy is unchanged even on increasing the ensemble size. On ignoring this outlier, the MUE reduces from 1.76 ± 0.32 to 1.30 ± 0.29 kcal/mol, and *r*_p_ increases from 0.57 ± 0.26 to 0.69 ± 0.20 kcal/mol when the ensemble size is increased from 5 to 10. This is an excellent example of how improved precision can be lead to better accuracy for flexible systems.

### 5.5 Choice of the Alchemical Region

The electrostatic interactions of atoms in the alchemical region are scaled such that in the intermediate λ windows they are very weak. This makes any charged group in the alchemical region very flexible, thus prone to high uncertainty and hence lower accuracy. PTP1B is an interesting case in point with all ligands containing two carboxylate groups in the active site. Moreover, one of them is attached to a thiophene ring through three rotatable bonds, and the binding pocket has enough empty space for it to freely move around. It has been discussed in more detail in the Supporting Information of our previous article.^14^ This provides a charged flexible group in PTP1B ligands which displays a predisposition for large fluctuations if included in the alchemical region. This is exactly what we find for 7 ligand pairs bound to PTP1B, where both these carboxylate groups constitute a part of the alchemical region chosen for our modified dual topology protocol using the standard charge tolerance criteria of 0.1e for both individual atoms as well as the entire common region. The standard error σ_TIES_ for these transformations varies between 1.4 and 2.3 kcal/mol with our standard protocol of 5 replicas per λ window.

**Figure 6:**
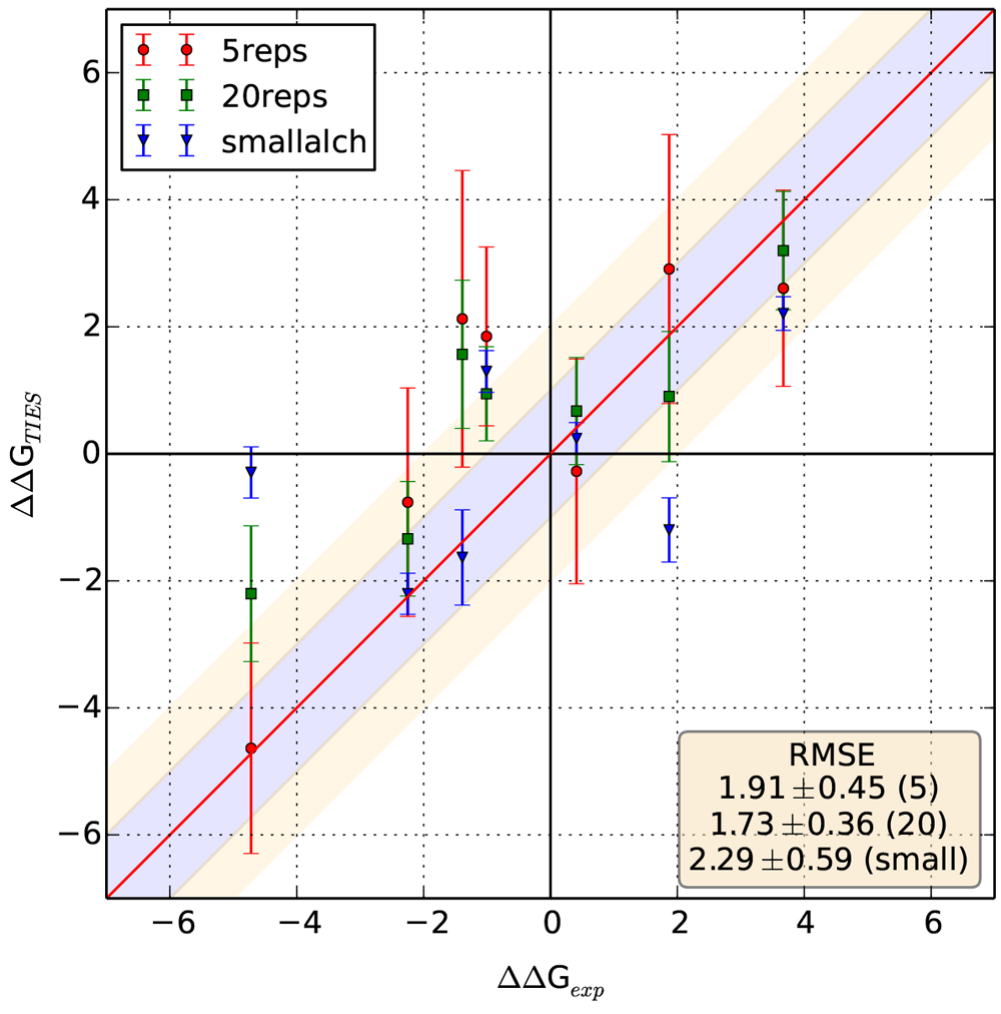
Comparison of results for 7 PTP1B ligand transformations that includes two charged carboxylate groups in the TIES alchemical region. Results with ensemble size 20 using the original alchemical region and ensemble size 5 using a smaller alchemical region (both carboxylates excluded) are compared with those from standard TIES. Shaded regions denote ±1 and ±2 kcal/mol ranges. Standard errors are included for all RMSE values. All values are in kcal/mol.

There are two routes to deal with this issue. First, one can increase the ensemble size in order to better control uncertainty, and second, one may relax the standard charge tolerance of 0.1e to reduce the size of the alchemical region so as to exclude the two carboxylate groups. Our results from both these approaches, compared with the standard one, are shown in Figure 6. As expected, the error bars for both are smaller than those for the standard protocol. σ_TIES_ falls in the range of 0.7–1.7 kcal/mol for ensemble size 20 using the same alchemical region, compared with 0.2–0.8 kcal/mol for the smaller alchemical region. Thus, TIES predictions using a smaller alchemical region (keeping ensemble size as 5) are more precise than those using ensemble size 20 (but the original alchemical region). However, relaxing the charge tolerance criteria (up to 0.14e for both individual atomic charges as well as overall MCS in this case) compromises the accuracy of the predictions. RMSE increases from 1.91 ± 0.45 to 2.29 ± 0.59 kcal/mol when using the smaller alchemical region. There are 2 clear outliers falling outside the ±2 kcal/mol range in Figure 6. This is despite the much higher precision of results with this approach. Therefore, this approach needs to be used cautiously.

On the other hand, increasing the ensemble size to 20 reduces RMSE to 1.73 ± 0.36 kcal/mol despite higher uncertainities on its predictions as compared to the alternative approach. Larger ensemble size improves accuracy for 6 out of 7 ligand pairs under consideration here. The only exception is the one where the standard protocol predicts ΔΔ*G* very close to the corresponding experimental value of −4.72 kcal/mol, whereas the large ensemble prediction falls outside the ±2 kcal/mol region. On ignoring this exceptional case, RMSEs for ensemble sizes 5 and 20 are 2.06 ± 0.46 and 1.56 ± 0.42 kcal/mol, respectively.

### 5.6 “Enhanced Sampling” Degrades Predictions

**Figure 7:**
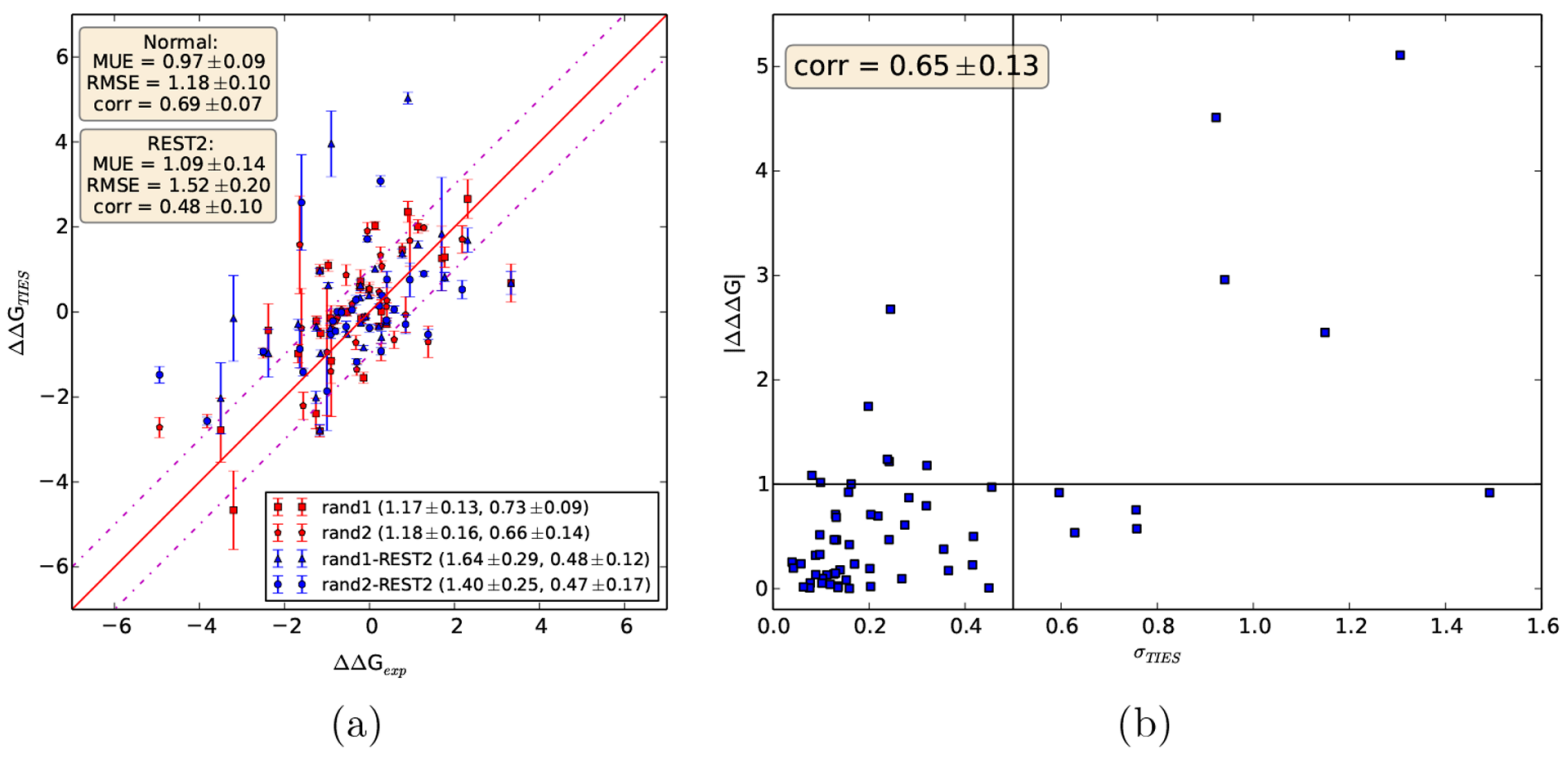
Degradation of results on employing the enhanced sampling method, REST2, when compared with normal MD simulations. (a) displays a comparison of ΔΔ*G*s obtained from both types of sampling for two mutually exclusive subsets of 30 randomly selected ligand pairs. The legend contains RMSEs followed by *r*_p_ values in brackets for each subset, whereas text boxes in the top left corner contain MUE, RMSE, and *r*_p_ values for the combined data set of 60 ligand pairs. (b) shows the variation of |ΔΔ*G*_REST2_ – ΔΔ*G*_normal_| with the corresponding uncertainties in ΔΔ*G* obtained without REST2 for all 60 ligand pairs. The vertical and horizontal lines correspond to σ = 0.5 kcal/mol and |ΔΔΔ*G*| = 1 kcal/mol, respectively. Standard errors are included for all reported values.

Replica exchange with solute tempering (REST2) is an enhanced sampling method that involves heating only a small region of the solute.^47^ Coordinates are exchanged periodically between replicas so as to allow easier crossing of energy barriers. It has been claimed that REST2 either improves free energy estimates or leaves them unchanged as compared to those obtained without REST2 (using normal MD simulations).^8^ Indeed, REST2 is supposed to allow sampling of less accessible states. However, as we have pointed out in previous studies, REST2 may lead to degradation in the accuracy of ΔΔ*G* predictions, and hence its uncritical use could be misleading.^18,22^ In this study, we have obtained consistent results with a statistically robust data set of 60 randomly selected ligand pairs from our full data set. We pulled out two mutually exclusive subsets of 30 randomly selected ligand pairs (denoted as “rand1” and “rand2”) and repeated TIES calculations with sampling “enhanced” using the REST2 protocol. The results are compared with those obtained using the standard TIES protocol (normal MD, that is, without REST2) in Figure 7(a). It is evident that REST2 degrades the accuracy of results for both rand1 and rand2, separately as well as for the combined data set of 60 ligand pairs. For rand1/rand2, RMSE increases from 1.17 ± 0.13/1.18 ± 0.16 to 1.64 ± 0.29/1.40 ± 0.25 kcal/mol, whereas *r*_p_ decreases from 0.73 ± 0.09/0.66 ± 0.14 to 0.48 ± 0.12/0.47 ± 0.17 kcal/mol. For the combined data set of 60 ligand pairs, MUE and RMSE increase by 0.12 and 0.34 kcal/mol, respectively, whereas *r*_p_ decreases from 0.69 ± 0.07 to 0.48 ± 0.10 kcal/mol. Thus, it can be concluded that blindly applying REST2 assuming that it will always have a positive impact and at worst none on the ΔΔ*G* predictions is wrong. Consequently, in drug discovery applications, one may risk excluding many potentially useful leads by simply applying REST2. Note that it is an inbuilt feature of the proprietary FEP+ package; an earlier study of ours comparing FEP+’s performance to TIES showed that the former generates worse results and degrades those further for longer simulation times.^22^ It should also be noted here that Khalak et al. reported degradation in accuracy of the predicted free energies using the PMX method on extending their simulations up to 1 μs.^27^

This apparently anomalous effect of REST2 may be explained by considering the fact that it involves biasing the potential so as to improve the sampling of otherwise less accessible states. However, this means that the weights of different accessed states of the ensemble are also biased. Ensemble averaging properties using such biased weights leads to inaccuracies in results. To further substantiate this argument, we have plotted the absolute differences between ΔΔ*G* predictions obtained with and without REST2 applied against the corresponding statistical uncertainties in standard TIES predictions without REST2 sampling, as shown in Figure 7(b). These two quantities are moderately correlated with *r*_p_ of 0.65 ± 0.13. Nine out of the 60 ligand pairs have uncertainties  kcal/mol. The MUE, RMSE, and *r*_p_ values for this subset with and without REST2 applied are 1.88 ± 0.55, 2.49 ± 0.58, and 0.47 ± 0.21 kcal/mol and 1.12 ± 0.31, 1.46 ± 0.37, and 0.75 ± 0.17 kcal/mol, respectively. Thus, the average deviation from normal MD results as well as the extent of corresponding degradation in accuracy are larger for this subset than those for the entire set of 60 ligand pairs (over a 6-fold increase in MUE; 0.76 versus 0.12 kcal/mol). This implies that, as the number of accessible states/minima (separated by relatively small energy barriers) for a system increases, the deviation of REST2 results from those obtained without it also increases, and so does the inaccuracy in results. It may be attributed to the higher bias introduced in weights due to the higher number of states accessible.

Another interesting observation is related to the precision of REST2 predictions as compared to those of normal MD predictions. It should be noted that REST2 predictions are more precise than normal MD predictions in general with lower σ_TIES_ for 53 out of the 60 ligand pairs studied. However, higher precision does not lead to better accuracy, as we have discussed a number of times.^18,22^ This is also true when the precision improves substantially. For instance, there are 3 ligand pairs for which σ_TIES_ reduces by more than 0.5 kcal/mol on applying REST2. However, 2 of them have higher unsigned errors for REST2 predictions than those for normal MD-based predictions. This suggests that replica simulations have closer distributions of conformations due to mixing of states caused by exchange of conformations in REST2. However, the biased weights are equally present in all replicas and hence lower the accuracy.

When focusing on the 7 ligand pairs with σ_TIES_ larger for REST2 predictions, another interesting trend is observed. Three of the 7 have σ_TIES_ increased by  kcal/mol which is insignificant and may be ignored. The remaining 4 belong to the BACE (scaffold) system which has all positively charged alchemical regions. As discussed earlier, charged groups in the alchemical region lead to large uncertainities and less accurate results with the standard TIES protocol due to attenuation of the electrostatic interactions possibly leading to more frequent sampling of higher energy conformations. This phenomenon is enhanced with REST2 due to further weakening of electrostatic interactions in the intermediate λ states. Thus, charged alchemical regions can be expected to have larger uncertainties when employing the REST2 protocol. In our data set of 60 ligand pairs, 5 have charged alchemical regions (all binding to the BACE (scaffold) system with a charge of +1). Four of them have larger uncertainties when using REST2 than without it. MUEs for these 5 BACE (scaffold) ligand pairs with and without REST2 are 2.74 ± 0.78 and 0.82 ± 0.21 kcal/mol, respectively, manifesting the degradation in accuracy on applying REST2 to this system. It should be noted here that all 5 of these ligand pairs have uncertainties ranging between 0.75 and 1.3 kcal/mol and hence form a subset of the 9 ligand pairs with σ_TIES_ > 0.5 kcal/mol as discussed earlier.

Thus, we recommend that the REST2 enhanced sampling method not be blindly applied. It is evident that the algorithm is not doing what its creators intended.

### 5.7 Effect of Transformation Size

**Figure 8:**
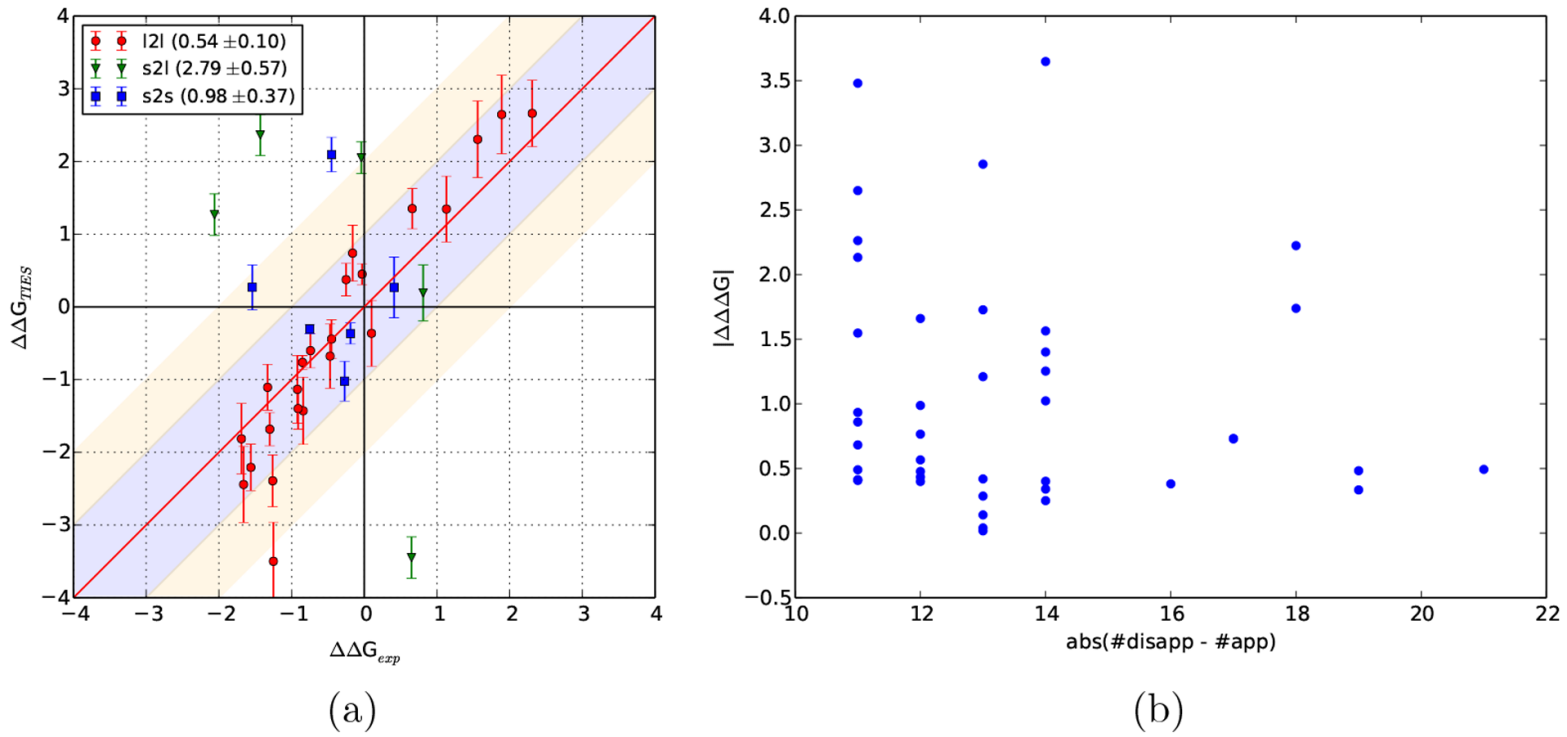
(a) Comparison of TIES results for PDE2 ligand transformations categorizing them into small to small (s2s; 6 transformations), large to large (l2l; 23 transformations), and small to large (s2l; 5 transformations). Corresponding MUEs along with associated standard errors are included in parentheses within the legend. All values are in kcal/mol. (b) Variation of unsigned errors with the size of transformation defined as the absolute difference between the number of disappearing and appearing atoms.

In a previous study using the PDE2 system, the ligands were classified as small or large based on the size of substituents attached to the scaffold.^57^ Ligands with a hydrogen or methoxy group as substituents were called “small”, and those with larger substituents were considered “large”. It was shown that FEP+ predicted ΔΔ*G* values for transformations from small-to-small (s2s) ligands, and large-to-large (l2l) ligands were relatively more accurate (MUEs less than 1 kcal/mol) compared to those from small-to-large (s2l) ligands (MUEs over 2 kcal/mol). We were able to reproduce this behavior in the present study with small MUEs for s2s and l2l transformations ( kcal/mol), whereas large MUEs for s2l transformations (2.79 ± 0.57 kcal/mol) are as shown in Figure 8(a).

To further investigate this issue, we pulled out some large transformations from our data set to see if the same behavior persists. To do so, we selected all transformations with the absolute difference between the number of disappearing and appearing atoms greater than 10, which amounts to 43 ligand pairs including 1 from the PDE2 set. We ignored the only transformation from the PDE2 data set, and the MUE for the remainder is 1.07 ± 0.14 kcal/mol. The MUE for the full data set (503 ligand pairs) is 1.04 ± 0.04 kcal/mol. Thus, the accuracy for “large” transformations is almost the same as that for the full data set. Figure 8(b) displays the variation of unsigned errors for these 42 transformations with the size of transformation (defined as the absolute difference between the number of disappearing and appearing atoms). There is no correlation between the two quantities which implies that the observed errors are independent of the size of the transformation. This indicates that the issue of less accurate predictions for large transformations is specific to the PDE2 system and is not a general one for alchemical methods.

Another interesting observation is the effect of the size of the alchemical region (defined as the number of atoms in the alchemical region) on the accuracy and precision of the predicted ΔΔ*G* values. As shown in a previous TIES study, the precision of predicted free energies decreases as the size of the alchemical region increases.^19^ On the other hand, the accuracy of such predictions remains relatively unaffected by this quantity.^19^ In the present study, we obtain consistent results. We pulled out all transformations with the sum of the appearing and disappearing atoms greater than 40 from the full data set (a total of 40 transformations with the sum ranging from 41 to 68) and compared their |ΔΔΔ*G*| as well as σ_TIES_ with the sum. The corresponding Pearson’s ρ for these quantities are 0.18 ± 0.15 and 0.45 ± 0.18, respectively, further confirming the previous findings.

### 5.8 Flexibility in Ensemble Size

Within the TIES protocol, we recommend flexibility in the ensemble size to be used depending on the level of precision desired and the system being investigated. Precision of results increases with ensemble size. However, the exact trend is system-specific, and so is the magnitude of uncertainty associated with the predicted ΔΔ*G* values. Therefore, a fixed ensemble size yields different error bars for different systems. This is evident in our current data set where 69 ligand pairs have uncertainties greater than 0.5 kcal/mol using ensemble size 5, while 81 are below 0.1 kcal/mol using the same ensemble size. We have already seen in the previous sections that increasing the ensemble size reduces uncertainty in predictions. This is why our protocol leaves the ensemble size parameter adjustable. However, this does not mean that the ensemble size can be arbitrarily small as that affects the accuracy of results. For instance, there are 70 ligand pairs in the current data set where σ_TIES_ is larger for ensemble size 5 compared to ensemble size 3. Similarly, on closely observing Figure 4(b), we can see prominent kinks for a few ligand pairs for ensemble sizes below 5. These observations indicate that, for a substantial number of systems, an additional replica beyond the second or third replica may represent a slightly different region of the phase space and hence increase associated uncertainty. Thus, using an ensemble size of only 2 or 3 does not capture the full picture, leading to overconfident predictions. In addition, as we have seen, the distribution of free energies deviates from Gaussian behavior; 2 or 3 replicas are simply insufficient to encompass contributions from heavy tails. One is unlikely to get the correct expectation value either, which is the quantity that determines accuracy.

The ensemble size, that is the number of replicas to be performed at each λ point, is the choice that needs further discussion as each added replica comes with additional computational cost (albeit no additional wall clock time is required on a supercomputer). So, one needs to find a trade-off between the computational cost and the level of precision (and consequently accuracy) desired, especially when the number of ligand pairs is large. For large-scale free energy calculations, a suggested way to employ TIES (or any other free energy method of choice) could be to decide a desired level of precision in terms of a threshold value of statistical uncertainty (σ). Thereafter, one can start with a “floor value” of ensemble size and perform calculations for all ligand pairs, followed by a stepwise increase in the ensemble size only for those ligands for which the resultant uncertainties are above the threshold σ. The larger ensemble size should only be required for a few systems where the ligands have flexible structure or charged groups in the alchemical regions (such as MCL1 and PTP1B cases discussed in sections 5.4 and 5.5). For instance, in our data set of 503 ligand pairs, over 86% achieved a precision of  kcal/mol using ensemble size 5. Such a progressive increase in ensemble size would ensure an optimal use of computational resources alongside achieving the desired level of precision for most calculations and resulting improvement in accuracy of results. One may choose the threshold σ to be 0.5 kcal/mol and the floor value of ensemble size to be 5.

### 5.9 Ligand Charge Methods

**Figure 9:**
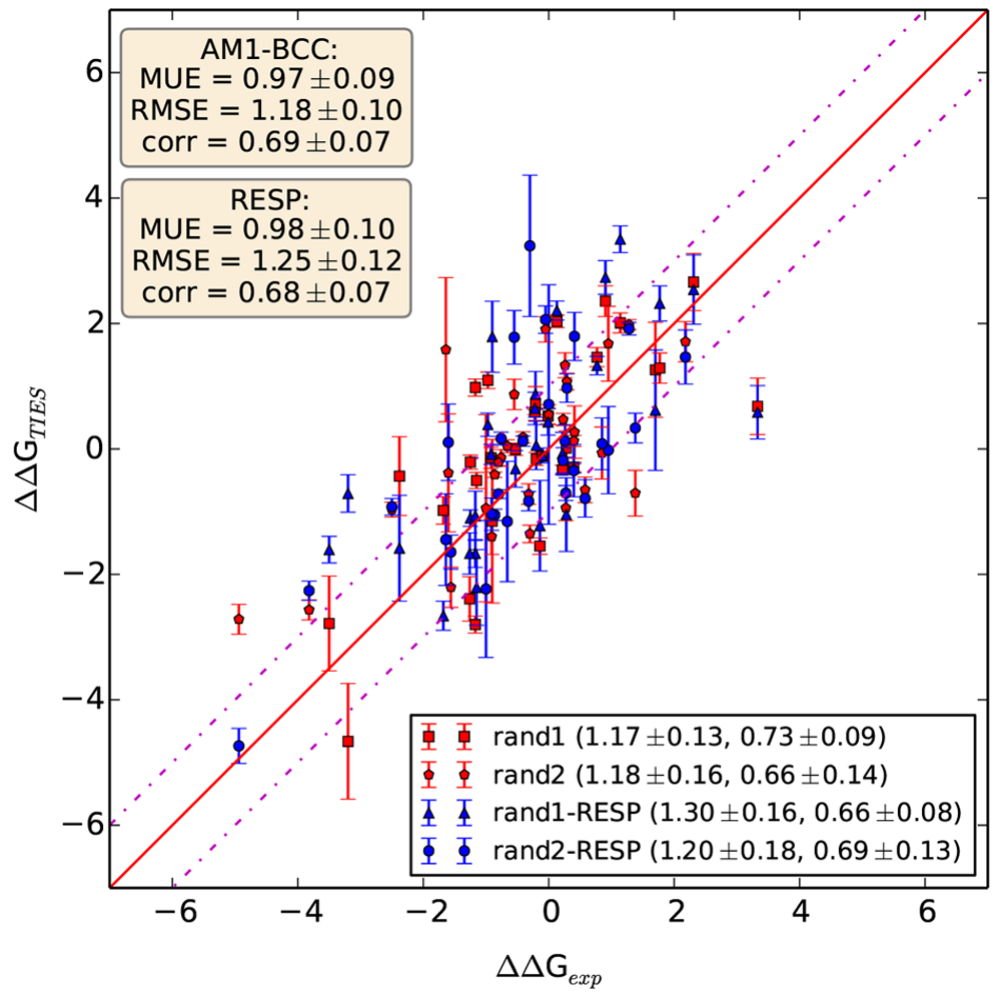
Comparison of results using AM1-BCC and RESP charge models for ligands. ΔΔ*G*s obtained using both charge methods for two mutually exclusive subsets of 30 randomly selected ligand pairs are shown. The legend at the lower right contains RMSEs followed by *r*_p_ values in brackets for each subset, whereas the legend in the top left corner contains MUE, RMSE, and *r*_p_ values for the combined data set of 60 ligand pairs along with corresponding standard errors.

In this study, we have compared ΔΔ*G* values obtained using RESP charges for ligands with those using AM1-BCC charges. For this, we use the same two mutually exclusive subsets of 30 randomly selected ligand pairs that have been used to study the effect of REST2. Figure 9 displays the results obtained using both these charge models. Overall, the performance is almost the same with MUEs and RMSEs differing only by 0.01 and 0.07 kcal/mol, respectively, and *r*_p_ differing by 0.01 for the combined set of 60 ligand pairs. When the two subsets are assessed separately, the performances are comparable for the “rand2” subset with RMSE and *r*_p_ slightly different for the two charge models. For the “rand1” subset, AM1-BCC charges perform slightly better in terms of RMSE and *r*_p_ than RESP. However, we cannot simply conclude that AM1-BCC charges are better than RESP charges based on this.

Another interesting fact is that RESP charges are more sensitive to chemical changes in ligand structures as compared to AM1-BCC charges. This is evident from the fact that, using the same charge tolerance criteria of 0.1e (difference between atomic charges on individual MCS atoms as well as the overall charge of MCS for the two ligands), the size of the alchemical region is larger for the RESP case as compared to AM1-BCC. The average number of disappearing atoms increases from 10.42 to 15.25 on using RESP charges for the 60 ligand pairs studied here. Similarly, the average number of appearing atoms also increases from 11.02 to 15.85 for all 60 ligand pairs. This trend is also visible for both subsets of 30 ligand pairs each separately. This need for fewer atoms in alchemical regions using AM1-BCC charges indicates that the effect of a given chemical change in a ligand is more localized with this charge model. On the other hand, the magnitude of change in RESP charges is more prominent for distant atoms. Although this higher sensitivity of RESP charges to chemical modifications in ligand molecules does not seem to affect the accuracy of results, it is bound to have an impact on their precision. Out of 60, 14 ligand pairs have σ_TIES_ ≥ 0.5 kcal/mol using RESP charges against only 9 using AM1-BCC charges. Similarly, the mean of σ_TIES_ for all 60 ligand pairs increases from 0.30 kcal/mol for AM1-BCC charges to 0.38 kcal/mol for RESP charges. Given that AM1-BCC-based models yield more precise results for a fixed amount of computation without compromising with their accuracy compared to RESP-based models and that it is much faster to compute charges using the AM1-BCC model, it should be preferred for free energy calculations.

## 6 Conclusion

This study presents ΔΔ*G* predictions using an open-source ensemble simulation-based alchemical free energy method for a large data set of 503 diverse ligand pairs bound to 14 different targets covering a broad range of protein classes and 305 different ligands. Such a detailed and systematic analysis of the various factors affecting RBFE predictions enables us to make definitive recommendations for the implementation of relative free energy calculations.

On comparing with the available experimental data, we achieve good agreement. However, we provide statistically reliable evidence for why standard metrics such as closeness to the experimental values and comparison with them using linear regression may not be reliable, particularly when the results are based on one-off or a few simulations. Such issues are alleviated by performing ensemble simulations in order to bring the substantial aleatoric uncertainty under control. Moreover, we have pointed out that the experimental predictions of free energies also have uncertainties associated with them and the nature of their distributions is generally non-normal, making a comparison of the calculated values with them unreliable.

We have confirmed that the distribution of free energies obtained from independent replica simulations covers a wide range of values and exhibits deviation from the Gaussian behavior usually assumed. This means that performing ensemble simulations is essential for such calculations. Another important consequence of the non-normal nature of such distributions is that the ensemble size cannot be arbitrarily small, and we recommend a minimum should comprise 5 replicas consistent with all our previous publications.^14,31^

We exploited the richness of our data set to perform systematic analyses on various important factors that affect the accuracy and/or precision of alchemical calculations. Flexible ligand structures and charged groups in the alchemical region lead to large fluctuations in the predicted free energies. The error bars in such cases can be controlled by increasing the ensemble size appropriately which also translates into improved accuracy of results. Such features substantially improve the predictive power of RBFE methods which is essential if they are to be actionable.

Using a statistically significant subset of the full data set studied, we compared the ΔΔ*G* predictions obtained with and without REST2, the enhanced sampling method, employed. We conclude that the REST2 protocol degrades the accuracy of results, and hence its routine application is not recommended. This might seem counterintuitive to those practitioners who assume that the worst REST2 could do is not affect the accuracy of results, but our findings raise serious concerns about the general validity of the REST2 protocol. Similarly, we found that the lightweight AM1-BCC charge model is able to achieve more precise results compared to the RESP charge model without compromising their accuracy and is thus preferable.

We have shown that the size of transformation does not affect the accuracy of results in general. We have used transformations with the absolute difference between the number of disappearing and appearing atoms up to 21 and found that the results are almost as accurate as those for the entire data set. Thus, the standard protocol of using 13 λ windows is sufficient for such large transformations. In addition, we have employed our modified dual topology scheme^14,19^ in this study, as it overcomes the issues associated with both single as well as dual topology schemes and combines the pros of them both. It has been successfully employed on a large data set in this study further confirming its general applicability. An online tool to automatically build models with this topology scheme for TIES calculations is freely available for anyone to use.^75^

Nonequilibrium methods based on MD are also known to exhibit dominant aleatoric uncertainties in the same way as equilibrium methods discussed in this study.^32−34^ Although nonequilibrium free energy methods such as PMX^26,79^ have not been investigated within this paper, our findings and previous studies indicate that it is necessary to perform similar systematic analyses for these methods too. By the same token, machine learning methods are strongly data-driven, and hence the underlying distributions of data must be accounted for in such methods to properly control the accuracy and/or precision of their predictions. These issues remain open for future research.

## 7 Data and Software Availability

The data underlying this study are available in the published article. All input structures and parameter files used in this study along with experimental and predicted ΔΔ*G* values and errors are available at https://github.com/UCL-CCS/LargeScaleTIES. Readers interested in rerunning NAMD simulations using our protocol with the input files provided should refer to https://www.ks.uiuc.edu/Research/namd/2.14/ug/.
